# Aqueous Solution-Processed Nanometer-Thin Crystalline Indium Ytterbium Oxide Thin-Film Transistors

**DOI:** 10.3390/nano12071216

**Published:** 2022-04-05

**Authors:** Wangying Xu, Chuyu Xu, Liping Hong, Fang Xu, Chun Zhao, Yu Zhang, Ming Fang, Shun Han, Peijiang Cao, Youming Lu, Wenjun Liu, Deliang Zhu

**Affiliations:** 1 College of Materials Science and Engineering, Shenzhen University, Guangdong Research Center for Interfacial Engineering of Functional Materials, Shenzhen Key Laboratory of Special Functional Materials, Shenzhen 518000, China; 13689541007@163.com (C.X.); hlp19920@163.com (L.H.); m.fang@szu.edu.cn (M.F.); hsdf52690@126.com (S.H.); pjcao@szu.edu.cn (P.C.); ymlu@szu.edu.cn (Y.L.); liuwj@szu.edu.cn (W.L.); 2 Shenzhen Key Laboratory of Ultraintense Laser and Advanced Material Technology, Center for Advanced Material Diagnostic Technology, and College of Engineering Physics, Shenzhen Technology University, Shenzhen 518118, China; 3 Department of Electrical and Electronic Engineering, Xi’an Jiaotong-Liverpool University, Suzhou 215123, China; chun.zhao@xjtlu.edu.cn; 4 Department of Electronic and Communication Engineering, Shenzhen Polytechnic, Shenzhen 518055, China; zhangyu18@szpt.edu.cn

**Keywords:** aqueous solution-processed, indium ytterbium oxide, ultra-thin, thin-film transistors, bias stress stability

## Abstract

We demonstrate the growth of ultra-thin (~5 nm) indium ytterbium oxide (In-Yb-O) thin film using a simple vacuum-free aqueous solution approach for the first time. The influences of Yb addition on the microstructural, chemical, optical, and electrical properties of In_2_O_3_ are well investigated. The analyses indicate that Yb dopant could suppress oxygen vacancy defects effectively owing to the lower standard electrode potential, lower electronegativity, and stronger metal-oxide bond strength than that of In. The optimized In-Yb-O thin-film transistors (TFTs) exhibit excellent electrical performance (mobility of 8 cm^2^/Vs and on/off ratio of ~10^8^) and enhanced stability. The triumph of In-Yb-O TFTs is owing to the high quality In_2_O_3_ matrix, the remarkable suppressor of Yb, and the nanometer-thin and atomically smooth nature (RMS: ~0.26 nm) of channel layer. Therefore, the eco-friendly water-induced ultra-thin In-Yb-O channel provides an excellent opportunity for future large-scale and cost-effective electronic applications.

## 1. Introduction

Metal oxide semiconductors have emerged as promising channel materials for next generation thin-film transistors (TFTs) because of their high mobilities, good optical transparency, low leakage currents, smooth surfaces, and large-scale uniformities [1,2,3]. Among the oxide semiconductors, indium oxide (In_2_O_3_) is of great interest due to its extremely high mobility, which enables advanced thin-film electronics [4,5,6,7,8]. Furthermore, high mobility (μ) In_2_O_3_ could be realized by solution processing, eliminating the need for costly and complex vacuum deposition techniques [3,9,10]. Unfortunately, it is difficult to control the high carrier density that is caused by the large amount of oxygen vacancies in pristine In_2_O_3_ TFTs. This leads to the deteriorations of overall performance, including high off current (I_off_), unacceptable negative threshold voltage (V_th_), large subthreshold swing (S), and poor device stability [4]. The pristine In_2_O_3_ TFTs with good properties often require some additional processes, such as adding passivation layer, ozone treatment, and atmospheric-pressure plasma (APP) treatment [11]. However, the uniformity and stability of the devices prepared by these processes still need to be improved. Doping with metal cations is one of the most efficient strategies to suppress oxygen vacancies and improve the electrical properties of In_2_O_3_ TFTs [10,12,13,14,15,16,17].

Previous studies indicate that the ideal dopant should have a lower electronegativity than In (1.78), lower standard electrode potential (SEP) than In (−0.34 V), and stronger metal-oxide dissociation energy than that of In-O (320 kJ/mol) [15]. The great difference in electronegativity between dopant and oxygen contributes to a strong metal-oxygen bond [18]. The SEP is considered as the main parameter to evaluate the ability of dopant to bind oxygen [19]. The metal-oxide dissociation energy is defined as the standard enthalpy change in reaction to the bond breaking. Hence, the principle of selecting ideal dopant is low electronegativity, low SEP, and high metal-oxide dissociation energy, which can effectively reduce the oxygen vacancy defects and improve the device performance and stability [20,21,22,23]. Yb is one of the most promising candidates with a low electronegativity of 1.20, low SEP of −2.19 V, and high metal-oxide dissociation energy of 387 kJ/mol [24]. Besides, the Yb and In share the same valence state of +3, which would not introduce additional electrons [16]. Meanwhile, Yb_2_O_3_ and In_2_O_3_ hold the same bixbyite structures and hence low defect densities could be expected [16,25]. Jun et al. reported the electrospun indium ytterbium oxide (In-Yb-O) nanofiber TFTs [26]. However, In-Yb-O thin-film channel material has not yet been demonstrated.

Here, we report the growth of nanometer-thin (~5 nm) In-Yb-O layers and their implementation in TFTs. The In-Yb-O thin film properties as a variation of Yb doping ratio were characterized by grazing incidence X-ray diffraction (GIXRD), atomic force microscopy (AFM), cross-sectional transmission electron microscopy (TEM), X-ray photoelectron spectroscopy (XPS), UV-vis spectroscopy, photoluminescence (PL), and electrical analysis. Moreover, the optimized In-Yb-O TFTs demonstrate enhanced electrical performance and bias stress stability. A further prominent asset is that the In-Y-O channel can be grown from aqueous solution through simple one-step spin coating, paving the way for future large-scale green manufacturing.

## 2. Materials and Methods

The In-Yb-O solutions were made by mixing In(NO_3_)_3_·xH_2_O and Yb(NO_3_)_3_·xH_2_O in DI water solvent. The total concentration of In-Yb-O solutions was fixed at 0.2 M with molar ratios of Yb/(In + Yb) of 0, 2, 5, 10, and 20 mol%, respectively. Heavily doped Si wafers with 100 nm thermally grown SiO_2_ were used as substrates. The In-Yb-O layers were accomplished by spin coating the precursors onto the oxygen plasma-treated Si/SiO_2_ wafers followed by annealing at 350 °C for 1 h. Finally, Al source/drain electrode arrays (W/L = 1500/100 μm, the large W/L ratio here could avoid mobility overestimation [3]) were deposited by thermal evaporation through shadow masks at room temperature to complete the In-Yb-O TFTs fabrication.

The morphology, crystal structure, and film thickness of In-Yb-O were analyzed by GIXRD, AFM, and cross-sectional TEM. The optical characteristics of In-Yb-O were investigated by PL and UV-vis spectroscopy. The chemical information of In-Yb-O was examined by XPS. The electrical characteristics of the In-Yb-O TFTs were investigated by a semiconductor parameter analyzer.

## 3. Results and Discussion

**Figure 1:**
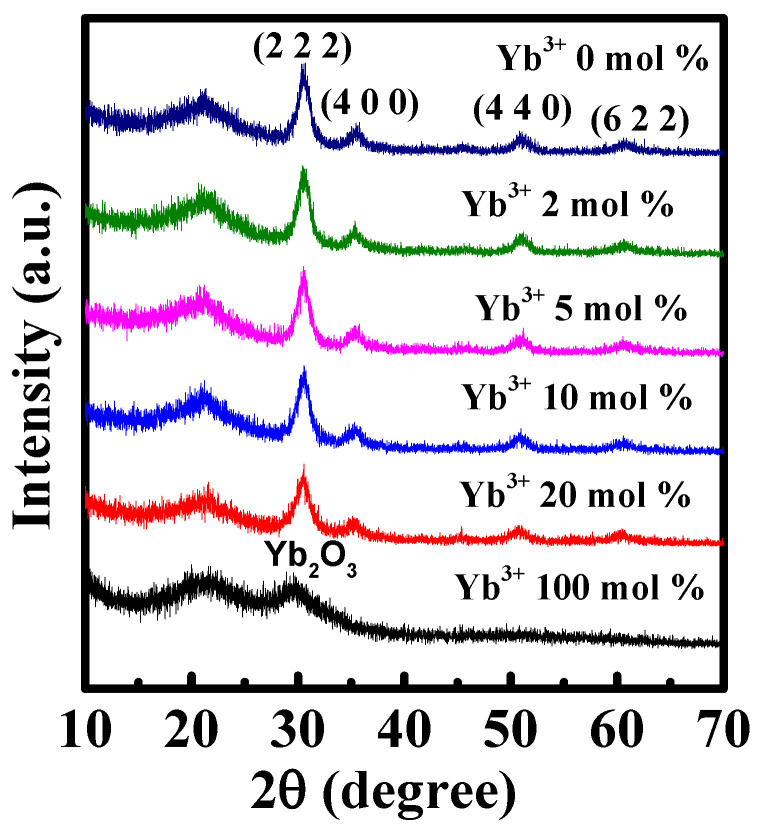
GIXRD patterns of the In-Yb-O with different Yb doping contents.

To understand the microstructure, optical bandgap and electrical characteristics of In-Yb-O channel material a comprehensive investigation was conducted and the relevant results are summarized in Table 1. Figure 1 demonstrates the GIXRD spectra of In-Yb-O thin films with different Yb ratios. The broad peaks in approximately 22–23° are attributed to the quartz substrate. In-Yb-O films are polycrystalline as suggested by the dominant (222) reflection at 30.580°, accompanied by weak (400), (440), and (622) crystal planes at 35.466°, 51.037°, and 60.676°. The XRD spectra of Yb_2_O_3_ are also measured, which shares the same bixbyite structure with In_2_O_3_. The XRD result indicates that Yb substitutes the In site of the In_2_O_3_ lattice. From XRD crystalline peak positions, the lattice constants of 0, 2, 5, 10, and 20% Yb-substituted In_2_O_3_ thin films are extracted to be 10.102, 10.120, 10.124, 10.127, and 10.159 Å, respectively [10]. It is noted that the extracted lattice constant of undoped In_2_O_3_ is in good accord with theoretical value (10.077 Å) [15], suggesting the validity of the calculation. The enhanced lattice constant after Yb incorporation is due to the fact that the ionic radius of Yb^3+^ (0.86 Å) is larger than that of In^3+^ (0.80 Å) [24]. The increase in lattice constant after Yb incorporation indicates the decline of In 5s orbital overlap, leading to the reduction in carrier concentration and device mobility [15], which will be discussed later.

**Figure 2:**
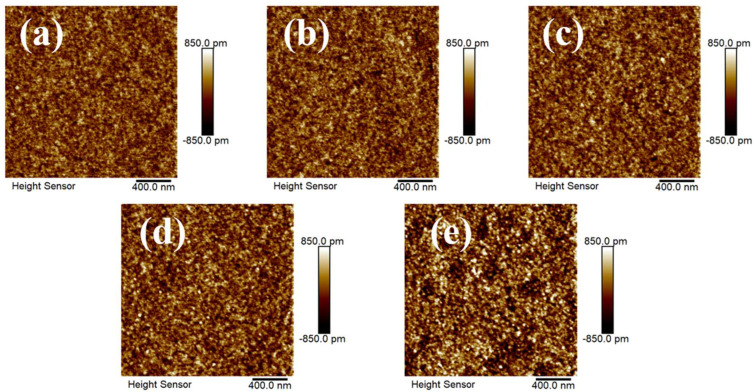
AFM images of In-Yb-O thin films with different Yb ratios of: (**a**) 0%, (**b**) 2%, (**c**) 5%, (**d**) 10%, and (**e**) 20%.

The surface morphologies of the In-Yb-O thin films were measured by AFM characterization as shown in Figure 2. All the In-Yb-O films demonstrate extremely smooth surfaces with RMS roughness of 0.225, 0.239, 0.248, 0.260, and 0.334 nm for the 0, 2, 5, 10, and 20% Yb doping contents, respectively. However, too much impurity addition will influence the film growth, and this is the case for slightly increased roughness for the 20% Yb-doped oxide thin film. The smooth channel topology is one of the key factors for achieving a high performance TFT device [3]. Besides, it is an unexpected result that crystalline oxide semiconductors with remarkable smooth surface could be fabricated by a facile aqueous route.

**Figure 3:**
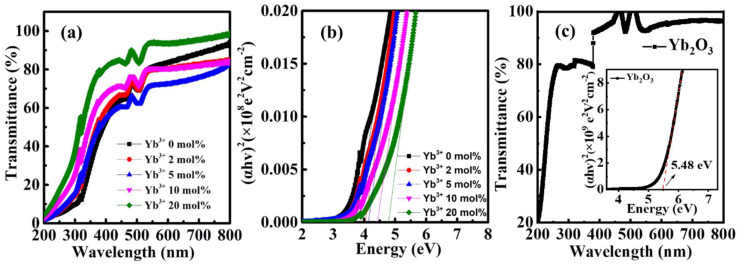
(**a**) Optical transmittance spectra, (**b**) estimated bandgaps of In-Yb-O thin films with indicated Yb ratios, and (**c**) optical transmittance spectra of Yb_2_O_3_ (inset: estimated bandgap of Yb_2_O_3_).

The optical characteristics of the Yb doped In_2_O_3_ thin films were accessed by UV-vis spectroscopy. As shown in Figure 3a, all the fabricated In-Yb-O thin films are highly transparent and the transparency increases with Yb doping ratio at short wavelength. The optical bandgaps of In-Yb-O thin films with 0, 2, 5, 10, and 20% Yb doping contents are determined to be 3.71, 4.04, 4.14, 4.43, and 4.78 eV, respectively (Figure 3b). In addition, the transparency and optical bandgap of Yb_2_O_3_ were also measured (Figure 3c). The Yb_2_O_3_ shows higher transparency at short wavelength and larger optical bandgap of 5.48 eV, in accord with previous research [25]. Hence, the increased optical bandgap with Yb incorporation is ascribed to the larger optical bandgap of Yb_2_O_3_ than that of In_2_O_3_.

**Figure 4:**
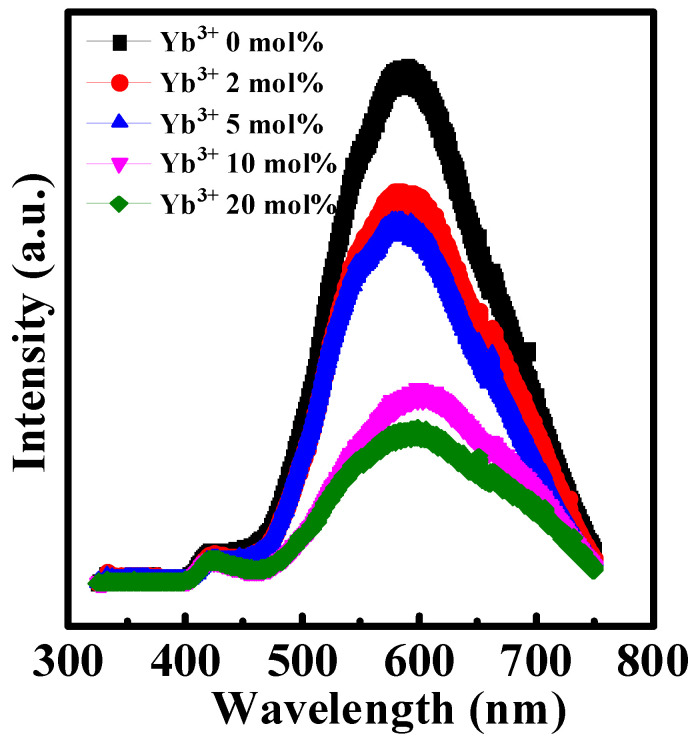
PL patterns of In-Yb-O with indicated Yb concentrations.

The PL patterns of Yb doped In_2_O_3_ thin films were shown in Figure 4. The dominant peak at ~590 nm could be observed. According to previous studies, this peak is ascribed to oxygen vacancy-related defects [20,27]. It is observed that the dominant peak intensity decreases with the rise of Yb doping ratio. Therefore, the Yb doping could reduce the oxygen vacancy-related defects in pristine In_2_O_3_.

**Figure 5:**
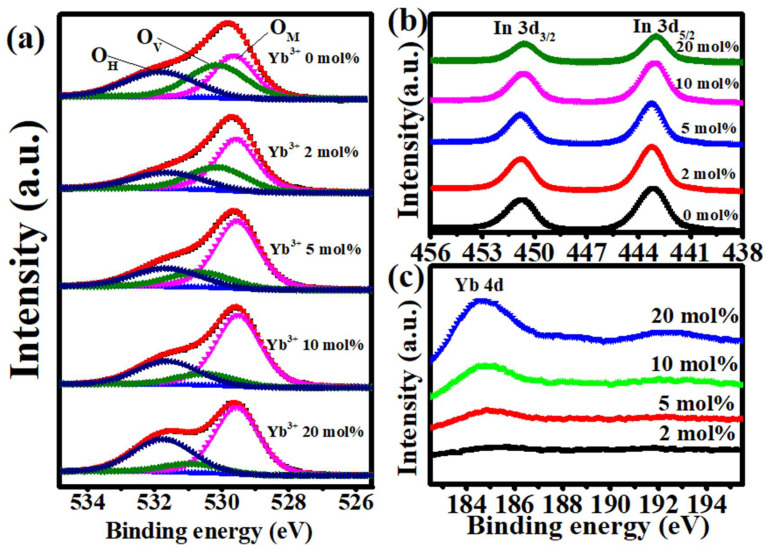
XPS spectra of: (**a**) O 1s, (**b**) In 3d, and (**c**) Yb 4d for In-Yb-O with different Yb ratios.

XPS was carried out to investigate the influence of Yb addition on the chemical state of the In_2_O_3_ film. Figure 5a demonstrates the XPS O 1s peak spectra of the In-Yb-O films. By using the Lorentz-Gaussian fitting method, the O 1s peak could be divided into three independent subpeaks of different oxygen environments, centered at binding energies of 529.62–529.67 eV (O_M_), 530.50–530.56 eV (O_V_), and 531.74–531.90 eV (O_H_). The O_M_, O_V_, and O_H_ peaks are related to oxide lattices (M-O-M), oxygen vacancies (V_O_), and metal hydroxide species (M-OH), respectively. As the doping amount of Yb increases from 0 to 20%, the O_M_/(O_M_ + O_V_ + O_H_) ratio increases from 34.13 to 64.38%, indicating that Yb can promote the formation of oxide lattices. As the Yb addition increases from 0 to 20%, the O_V_/(O_M_ + O_V_ + O_H_) ratio falls from 35.29 to 9.48%. The result shows that the Yb acts as an effective O_V_ suppressor as a consequence of stronger Yb-O bonding (387 kJ/mol) than that of In-O (346 kJ/mol). Besides, the increased O_H_/(O_M_ + O_V_ + O_H_) ratio with Yb, indicating the incomplete dehydration of residual Yb(OH)_3_. The XPS spectra of In 3d peaks of In-Yb-O thin films with different Yb ratios were shown in Figure 5b. The In peaks located at binding energies of 451.4 eV (In 3d_3/2_) and 443.9 eV (In 3d_5/2_) suggest the presence of In-O bonding [19]. With the Yb ratio increases, the In peak shifts toward a lower binding energy direction owing to stronger Yb-O binding strength (387 kJ/mol) than that of In-O (320 kJ/mol) [17]. The XPS spectra of Yb 4d peaks are shown in Figure 5c. The binding energy at 184.9 eV is related to the Yb_2_O_3_ reference position [25], indicating the formation of Yb-O bonding. The increase in Yb peak intensity with Yb precursor doping ratio could be observed, indicating that Yb incorporation into the In-Yb-O thin films.

**Figure 6:**
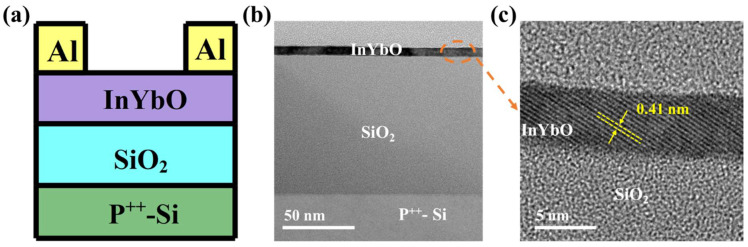
(**a**) Schematic architecture, (**b**) cross-sectional TEM image of the In-Yb-O (10% Yb) device, and (**c**) HRTEM image of selected area marked with a red circle in panel b.

**Figure 7:**
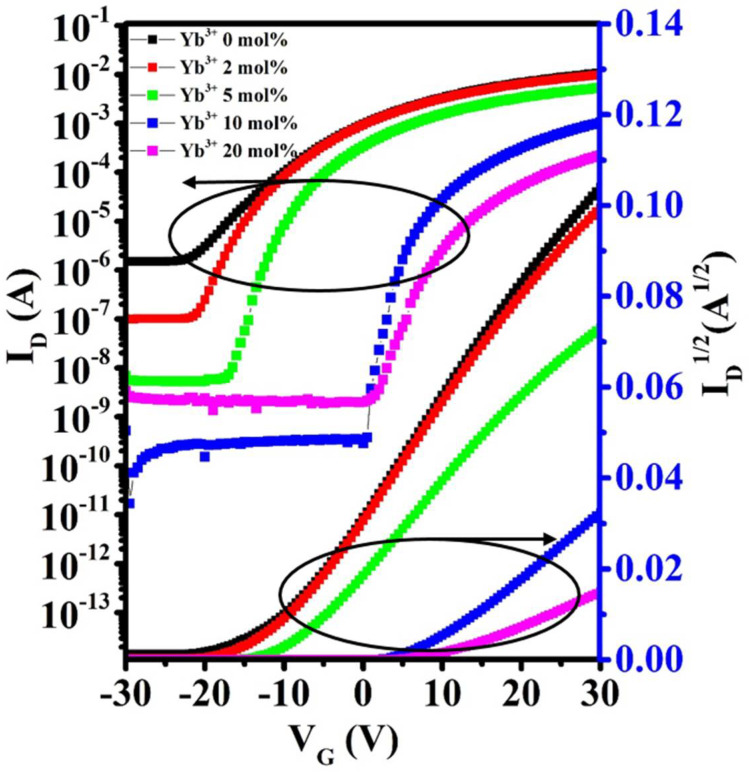
Representative transfer characteristics of In-Yb-O TFTs with different Yb doping contents.

**Figure 8:**
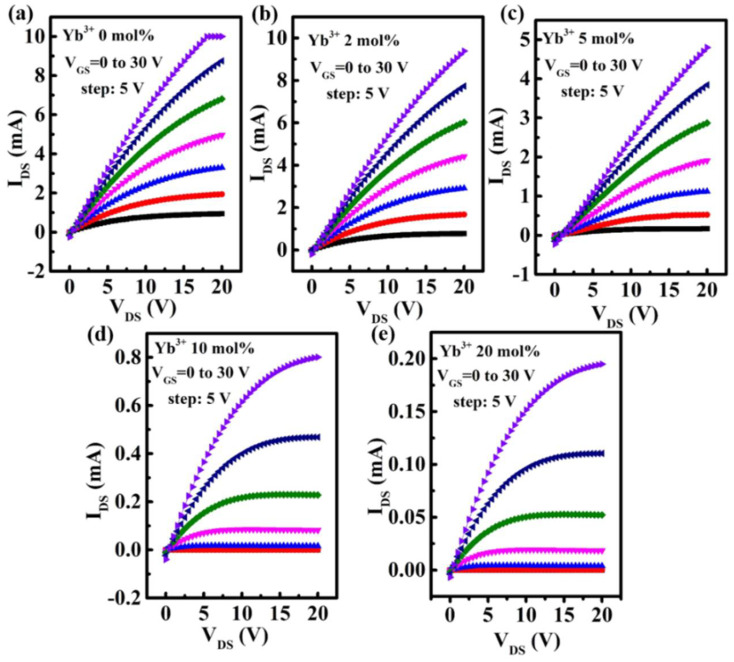
Output characteristics of In-Yb-O TFTs with Yb doping ratios of: (**a**) 0%, (**b**) 2%, (**c**) 5%, (**d**) 10%, and (**e**) 20%.

**Figure 9:**
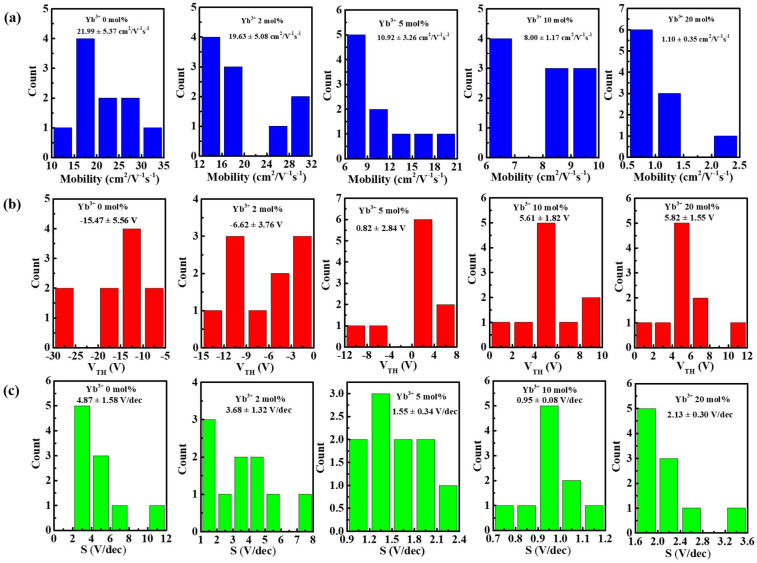
Histogram of: (**a**) mobility, (**b**) threshold voltage, and (**c**) subthreshold slope for the In-Yb-O TFTs with indicated Yb doping ratios.

Figure 6a shows the schematic diagram of In-Yb-O TFT with a bottom gate and top contact architecture. To verify the microstructural characteristics of the In-Yb-O device, cross-sectional TEM characterization was performed. Figure 6b exhibits a well-defined and highly uniform In-Yb-O layer with thickness of ~5 nm. The high-quality contact between In-Yb-O channel and SiO_2_ dielectric would guarantee low interface defect states and good current modulation. We can roughly estimate whether the contact is good by both TEM image and the interface trap density (calculated via subthreshold swing of the TFT device [20]). Figure 6c shows a high-resolution transmission electron microscope (HRTEM) image, in which the atoms are arranged in order and the surface is very neat. The interplanar spacing from HRTEM is about 0.41 nm, corresponding to the (211) crystal plane of cubic structure In_2_O_3_. The peak position about (211) crystal plane in XRD spectrum is at ~21°, which may be covered by the broad peak of quartz substrate and hence not observed. The GIXRD, HRTEM and AFM results suggest that even when crystalline, the In-Yb-O films do no exhibit large grains or prominent grain boundaries. In Smith’s research, it was also found that the spin-coated In_2_O_3_ film was nanocrystals with smooth a surface (RMS: ~0.2 nm) [22]. Another similar result can be found by Song et al. [18]. To investigate the role of Yb doping, the electrical properties of In-Yb-O TFTs were measured. Typical transfer and output plots as well as device statistics (10 TFTs) and average electrical parameters (μ, I_on_ /I_off_, V_th_, and S) are shown in Figure 7, Figure 8 and Figure 9 and Table 2. Note that pristine In_2_O_3_ TFTs show high μ of 21.99 cm^2^/Vs but suffer from poor I_off_ of ~10^−7^ A and unaccepted V_th_ of −15.47 V, which is not applicable for switching. The high I_off_ and large negative V_th_ are attributed to excess carrier concentration originated from the existence of a large amount of oxygen vacancies in pristine In_2_O_3_. Yb holds a lower SEP (−2.19 V), lower electronegativity (1.2), and stronger metal-oxide bond strength (387 kJ/mol) than that of In [24]. Thus, the oxygen vacancy defects in In_2_O_3_ could be effectively suppressed after Yb addition, leading to a great reduction in I_off_ (from 10^−7^ to ~10^−11^ A) and positive shift of V_th_ (from −15.47 to 5.82 V). Meanwhile, μ falls from 21.99 to 1.10 cm^2^/Vs as Yb ratio increases to 20%, which is attributed to the reduction of In 5s orbitals overlap after Yb incorporation [15]. In addition, the S value also decreases from 4.87 to 0.95 V/decade when Yb ratio increases to 10%. The improvement of S suggests the reduction in semiconductor/insulator interface trap states also associated with O_V_ related defects. However, too much Yb (20%) incorporation would deteriorate the subthreshold slope, which may be due to the incomplete dehydration of residual Yb(OH)_3_ [4]. As described in Figure 8, the linear increase in the output curves at low V_DS_ indicates good ohmic contacts between the In-Yb-O active layer and the Al source/drain electrodes. The thickness of In-Yb-O reported in this study is 5 nm. The transfer curves for 10% Yb doped In-Yb-O TFTs with different thicknesses are shown in Figure S1, with electrical parameters summarized in Table S1. The In-Yb-O TFTs with 10% Yb yield has the best overall electrical performance, with µ of 8.00 ± 1.17 cm^2^/Vs, S of 0.95 ± 0.08 V/decade, V_th_ of 5.61 ± 1.82 V, and I_on_/I_off_ of 6.19 × 10^7^, respectively.

**Figure 10:**
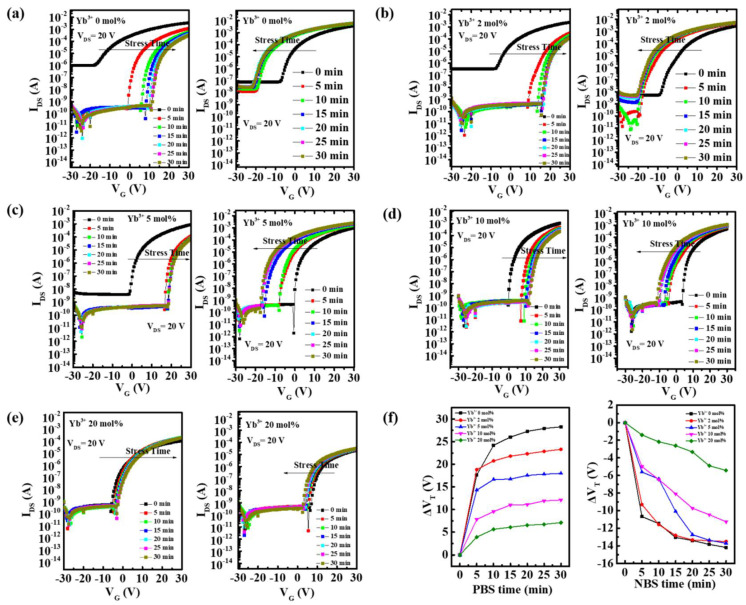
Transfer curves variations under PBS and NBS of In-Yb-O TFTs with Yb ratios of: (**a**) 0%, (**b**) 2%, (**c**) 5%, (**d**) 10%, and (**e**) 20%. (**f**) Summary of V_th_ shifts under PBS and NBS.

The electrical stabilities of In-Yb-O TFTs with different Yb ratios were also studied. Figure 10 demonstrates the transfer curves variations of In-Yb-O devices under positive-bias-stress (PBS) of 20 V and negative-bias-stress (NBS) of −20 V for 30 min. For all the In-Yb-O devices, positive V_th_ shifts under PBS could be observed, which could be ascribed to the electron trapping at the In-Yb-O or In-Yb-O/SiO_2_ interface [3]. Due to the suppression of the O_V_, Yb incorporation could enhance the PBS stability. Besides, because the In-Yb-O TFTs are without encapsulation, the adsorption of oxygen from ambient could also contribute greatly to the V_th_ variation under PBS [10]. On the other hand, In-Yb-O devices showed negative V_th_ shifts induced by NBS. The negative V_th_ shift is generally ascribed to the release of electrons from donor-like traps related with O_V_ [28]. Hence the NBS stability is also improved with the addition of Yb. In addition, the adsorption of water from ambient also leads to the negative V_th_ shifts.

The triumph of In-Yb-O (10% Yb) TFTs is owing to several factors. First, the high mobility of the In-Yb-O device is owing to the high quality In_2_O_3_. In the current research, by introducing the novel aqueous solution processing, nanocrystalline In_2_O_3_ TFTs with high mobility (22 cm^2^/Vs) could be realized. Secondly, the undoped In_2_O_3_ device suffers from poor I_off_, V_th_, and S due to the existence of excess O_V_, thus the incorporation of a suitable inhibitor is required. Yb is an ideal dopant with a low SEP, low electronegativity, and strong Yb-O dissociation energy. Yb_2_O_3_ and In_2_O_3_ have the same bixbyite structures and thus afford low defect states. Meanwhile, the Yb and In hold the same valence state (+3), so the introduction of Yb would not generate additional electrons. Thirdly, we would like to emphasize the benefits of our aqueous metal-nitrate precursors. Compared with a conventional organic solvent, water is an ideal choice since it contains no impurities. In addition, metal-nitrate precursors have proved superior to other salts due to their high volatility of decomposition byproducts [29]. Special hexaaqua metal complexes (In(H_2_O)_6_ or Yb(H_2_O)_6_) are formed in aqueous solution, which ensures the formation of dense and impurity-free metal oxide at a low processing temperature [9]. Consequently, by carefully optimizing the precursor solution concentration, an ultra-thin In-Yb-O channel with nanoscopically dense nature and extremely smooth surface could be produced. The nanometer-thin nature of In-Yb-O means a low density of the defect states in the transistor channel. The atomically smooth In-Yb-O/SiO_2_ interface would guarantee the ultra-fast electrons transport pathways. Besides, solution processing makes the composition tuning much easier than conventional vacuum-based deposition methods. The combined factors afford the solution-phase grown In-Yb-O TFTs with superior switching characteristics.

## 4. Conclusions

In summary, we report an efficient aqueous solution route to produce nanometer-thin (~5 nm) crystalline In-Yb-O channel material. By optimizing the Yb doping ratio, oxygen vacancies related defects could be suppressed, affording high performance In-Yb-O TFTs with mobility of 8 cm^2^/Vs and on/off ratio of ~10^8^. The combined analyses indicate that the success of In-Yb-O is owing to the crystalline In_2_O_3_ matrix, the superior dopant of Yb, and the nanometer-thin, nanoscopically dense nature of the channel layer as well as atomically smooth In-Yb-O/SiO_2_ interface. Furthermore, since Young’s modulus is inversely proportional to the thin-film thickness, the nanometer-thin In-Yb-O channel can ensure high mechanical ductility in flexible substrate. Therefore, the water-induced nanometer-thin In-Yb-O TFTs hold great potential for large-area electronics.

## Figures and Tables

**Table 1:** RMS roughness, lattice constant, and optical bandgap of In-Yb-O thin films with different Yb ratios.

Yb Ratios (%)	RMS (nm)	Lattice Constant (Å)	E_g_ (eV)
0	0.225	10.102	3.71
2	0.239	10.120	4.04
5	0.248	10.124	4.14
10	0.260	10.127	4.43
20	0.334	10.159	4.78

**Table 2:** Electrical performance parameters of In-Yb-O TFTs with different Yb ratios.

**Yb Ratio (%)**	μ	I_on_/I_off_	S (V/dec)	V_th_ (V)
0	21.99 ± 5.37	7.24 × 10^3^	4.87 ± 1.58	−15.47 ± 5.56
2	19.63 ± 5.08	9.87 × 10^4^	3.68 ± 1.32	−6.62 ± 3.76
5	10.92 ± 3.26	1.01 × 10^6^	1.90 ± 0.81	0.82 ± 2.84
10	8.00 ± 1.17	6.19 × 10^7^	0.95 ± 0.08	5.61 ± 1.82
20	1.10 ± 0.35	1.66 × 10^5^	2.13 ± 0.30	5.82 ± 1.55

## Supplementary Materials

The following are available online at https://www.mdpi.com/article/10.3390/nano12071216/s1, Figure S1: Transfer curves for 10% Yb doped In-Yb-O TFTs with different thicknesses, Table S1: Summary of the electrical characteristics of 10% Yb doped In-Yb-O TFTs with different thicknesses.
