# Supplementary material for: Aqueous Solution-Processed Nanometer-Thin Crystalline Indium Ytterbium Oxide Thin-Film Transistors
Source: Nanomaterials (Basel). 2022 Apr 5;12(7):1216. doi: 10.3390/nano12071216 (PMC9000645; doi:10.3390/nano12071216)
Supplement: Supplementary file 1 [file nanomaterials-12-01216-s001.zip › nanomaterials-1645215-supplementary.pdf]

Supplementary Material

Aqueous Solution-Processed Nanometer-Thin Crystalline Indium Ytterbium Oxide Thin-Film Transistors

Wangying Xu [1,*], Chuyu Xu [1], Liping Hong [1], Fang Xu [2,*], Chun Zhao [3], Yu Zhang [4], Ming Fang [1], Shun Han [1], Peijiang Cao [1], Youming Lu [1], Wenjun Liu [1] and Deliang Zhu [1,*]

1 College of Materials Science and Engineering, Shenzhen University, Guangdong Research Center for Interfacial Engineering of Functional Materials, Shenzhen Key Laboratory of Special Functional Materials, Shenzhen 518000, China; 13689541007@163.com (C.X.); hlp19920@163.com (L.H.); m.fang@szu.edu.cn (M.F.); hsdf52690@126.com (S.H.); pjcao@szu.edu.cn (P.C.); ymlu@szu.edu.cn (Y.L.); liuwj@szu.edu.cn (W.L.)
2 Shenzhen Key Laboratory of Ultraintense Laser and Advanced Material Technology, Center for Advanced Material Diagnostic Technology, and College of Engineering Physics, Shenzhen Technology University, Shenzhen 518118, China
3 Department of Electrical and Electronic Engineering, Xi'an Jiaotong-Liverpool University, Suzhou 215123, China; chun.zhao@xjtlu.edu.cn
4 Department of Electronic and Communication Engineering, Shenzhen Polytechnic, Shenzhen 518055, China; zhangyu18@szpt.edu.cn
* Correspondence: wyxu@szu.edu.cn (W.X.); xufang@sztu.edu.cn (F.X.); dlzhu@szu.edu.cn (D.Z.)

1. Detail Analysis of the Advantages of Yb Dopant.

The following principles are taken into account when selecting the appropriate dopant. First, there needs to be a significant difference in electronegativity between the dopant and the oxygen, which helps form strong metal-oxygen bonds[1]. Secondly, the standard electrode potential is an important parameter to measure the bonding ability of the dopant to oxygen. The lower the standard electrode potential is, the easier it is to combine with oxygen and reduce oxygen vacancy (V_o)[2]. Thirdly, the metal oxide bonding strength of the dopant must be stronger than that of the host materials to improve the stability of TFTs [3]. Besides, the Yb and In share the same valence state of +3, which would not introduce additional electrons [4]. Meanwhile, Yb_2O_3 and In_2O_3 hold the same bixbyite structures and hence low defect densities could be expected [4,5] Based on the above considerations, Yb dopant can effectively reduce the oxygen vacancy concentration in thin film, and realize the high performance In-Yb-O thin film transistor.

2. The Electrical Characteristics of In-Yb-O TFTs with Different Thicknesses.

The thickness of thin film is very important for solution-based oxide TFTs. The thickness of In-Yb-O reported in this study is 6 nm. The transfer curves for 10% Yb doped In-Yb-O TFTs with different thicknesses are shown in Figure S1, with electrical parameters summarized in Table S1. In-Yb-O TFTs with thickness of ~6 nm demonstrate the best performance.

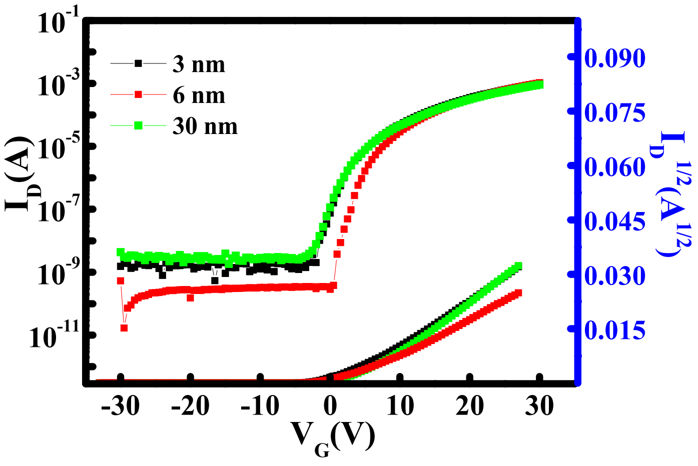

Figure S1. Transfer curves for 10% Yb doped In-Yb-O TFTs with different thicknesses.

Table S1. Summary of the electrical characteristics of 10% Yb doped In-Yb-O TFTs with different thicknesses.

Thickness (nm)	μ ($cm^2V^{-1}s^{-1}$)	I_{on}/I_{off}	SS (V/dec)	V_{th} (V)
3	7.39	1.91×10^6	1.05	2.31
5	8.00	1.09×10^8	0.95	−5.61
30	6.63	4.97×10^5	1.58	−1.15

References

1. Song, W.; Lan, L.; Li, M.; Wang, L.; Lin, Z.; Sun, S.; Li, Y.; Song, E.; Gao, P.; Li, Y.; et al. High-performance thin-film transistors with solution-processed ScInO channel layer based on environmental friendly precursor. *J. Phys. D: Appl. Phys.* **2017**, *50*, 385108.
2. Ting, C.C.; Fan, H.Y.; Tsai, M.K.; Li, W.Y.; Yong, H.E.; Lin, Y.F. Improvement of electrical characteristics in the solution-processed nanocrystalline indium oxide thin-film transistors depending on yttrium doping concentration. *Phys. Status Solidi A* **2014**, *211*, 800.
3. Luo, Y.-R.; Luo, Y.-R. *Comprehensive Handbook of Chemical Bond Energies*; CRC Press: Boca Raton, FL, USA, 2007; 1655 p.
4. Lin, Z.; Lan, L.; Sun, S.; Li, Y.; Song, W.; Gao, P.; Song, E.; Zhang, P.; Li, M.; Wang, L.; et al. Solution-processed high-mobility neodymium-substituted indium oxide thin-film transistors formed by facile patterning based on aqueous precursors. *Appl. Phys. Lett.* **2017**, *110*, 133502.
5. Pan, T.-M.; Huang, W.-S. Effects of Oxygen Content on the Structural and Electrical Properties of Thin Yb[sub 2]O[sub 3] Gate Dielectrics. *J. Electrochem. Soc.* **2009**, *156*, G6.
